# Getting to S: CDK functions and targets on the path to cell-cycle commitment

**DOI:** 10.12688/f1000research.9463.1

**Published:** 2016-09-26

**Authors:** Robert P. Fisher

**Affiliations:** 1 Department of Oncological Sciences, Icahn School of Medicine at Mount Sinai, New York, NY, USA

**Keywords:** cell cycle, cell cycle checkpoints, CDKs, cyclin-dependent kinases, G1 progression, S-phase entry

## Abstract

How and when eukaryotic cells make the irrevocable commitment to divide remain central questions in the cell-cycle field. Parallel studies in yeast and mammalian cells seemed to suggest analogous control mechanisms operating during the G1 phase—at Start or the restriction (R) point, respectively—to integrate nutritional and developmental signals and decide between distinct cell fates: cell-cycle arrest or exit versus irreversible commitment to a round of division. Recent work has revealed molecular mechanisms underlying this decision-making process in both yeast and mammalian cells but also cast doubt on the nature and timing of cell-cycle commitment in multicellular organisms. These studies suggest an expanded temporal window of mitogen sensing under certain growth conditions, illuminate unexpected obstacles and exit ramps on the path to full cell-cycle commitment, and raise new questions regarding the functions of cyclin-dependent kinases (CDKs) that drive G1 progression and S-phase entry.

## Introduction

Cell division is an intrinsically cyclical process in which cells alternately duplicate their genetic material and distribute the copies equally to identical daughters, potentially *ad infinitum*. Nevertheless, the G1 phase, prior to the decision to begin DNA replication in S phase, has historically been regarded as the “beginning” of the cell cycle, when cell-fate decisions are made—to divide or not to divide, or to differentiate, which usually entails cell-cycle exit. Implicit in the concept of a stringently regulated cell-cycle commitment is the possibility that such regulation might be weakened or lost owing to genetic or environmental factors, potentially leading to the inappropriate cell-division decisions associated, in multicellular organisms, with cancer.

A restriction (R) point in the mammalian cell cycle was operationally defined over four decades ago in cells emerging from quiescence as the point at which further progression became independent of continued mitogenic stimulation ^1^. A similar transition could be detected during G1 phase in continuously cycling cells ^2^, reinforcing the notion that mitogen sensing and commitment were inextricably linked and occurred in a narrow time interval “early” in the division cycle. Over the ensuing years, molecular mechanisms underlying these phenomena have been revealed, but questions have arisen regarding the precise nature and physiologic significance of the R point. Here I will review these developments and highlight instances in which classical studies of cell-cycle commitment might need to be reconciled with recent analyses by more modern techniques.

## A transcription switch synonymous with the R point?

**Figure 1.**
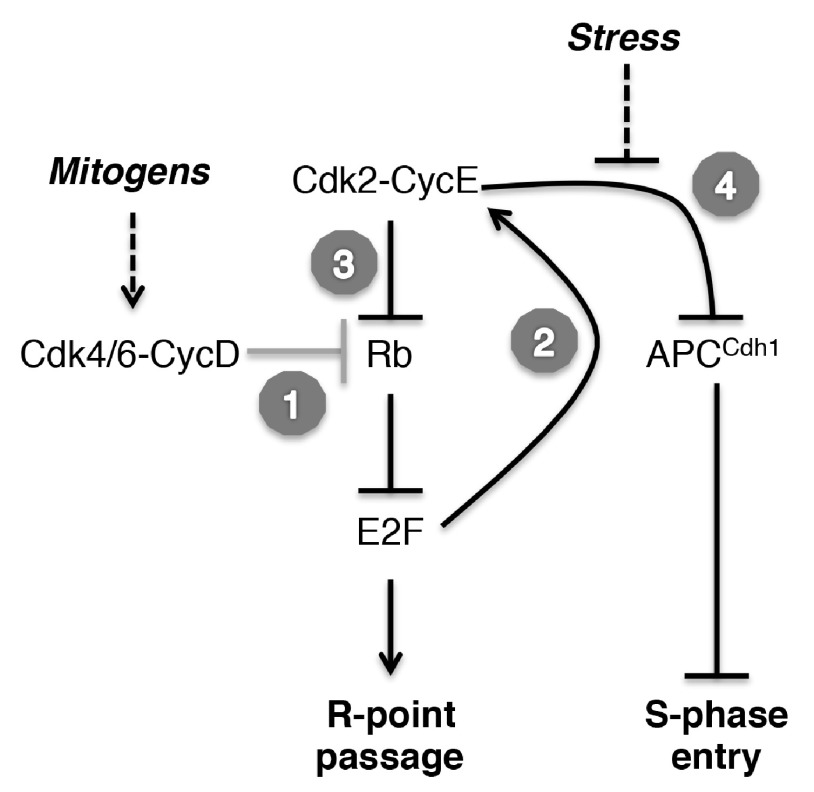
An updated model of cell-cycle commitment steps. In the classical view of restriction (R) point passage, cyclin D-dependent kinases (Cdk4 and Cdk6) are activated in response to mitogenic signaling and phosphorylate Rb to partially relieve the repression of E2F-dependent gene expression ( *step 1*). Among the first genes activated by E2F1 ( *step 2*) is the one encoding cyclin E ^11^, which activates Cdk2 to complete the phosphorylation of Rb ( *step 3*), fully activating the E2F program and promoting passage of the R point. This commitment occurs during G0-G1 progression in cells exiting quiescence but may be executed during the prior G2-M interval in continuously dividing cells in culture ^16, 18^. Cdk2 also phosphorylates and inactivates APC ^Cdh1^ ( *step 4*) to remove the last barrier to S-phase entry ^20^. Intercurrent stresses (e.g. DNA damage) can block this step and cause reversion to a mitogen-sensitive, quiescent state, but only while APC ^Cdh1^ remains active. The dual requirement for Cdk2 activity—at R-point passage and S-phase entry—was detected by a chemical-genetic analysis in human cells that excluded contributions by other kinases ^19^.

The R point bears more than a superficial resemblance to Start, when yeast cells become refractory to G1 cell-cycle arrest induced by nutrient deprivation or pheromones and will complete an entire round of cell division in the presence of mating factors before arresting during the next G1 ^3^. There are also parallels between yeast and metazoan G1 control at the mechanistic level, both in the upstream signaling components—the cyclin-dependent kinases (CDKs) that drive progression through the cell cycle—and the G1/S effectors they regulate. In both cases, sequential activation of distinct CDK-cyclin complexes leads to stepwise activation of a transcriptional program controlled by heterodimeric activators and associated, phosphorylation-sensitive repressors (reviewed in 4). The similarities run deep: the first CDK-cyclin complex to become active in G1 (Cln3-Cdk1 in budding yeast or cyclin D-Cdk4/6 in metazoans) promotes initial phosphorylation of the repressor (yeast Whi5 or metazoan pocket proteins of the retinoblastoma, or Rb, family) to induce transcription dependent on the activators (yeast SBF and MBF or metazoan E2F-DP isoforms). The transcriptional targets of these activators include additional G1 cyclins that form complexes with a CDK (yeast Cln1/2-Cdk1 or metazoan cyclin E-Cdk2), which fully activate G1/S transcription through a positive feedback loop ^5^ ( Figure 1). Whereas the CDKs and cyclins are broadly conserved, the transcriptional activators and repressors are not (reviewed in 6). A recent evolutionary analysis suggested that the last common ancestor of metazoans, plants, and fungi contained Rb and E2F orthologs; SBF/MBF and Whi5 orthologs acquired early in evolution of the fungal lineage by horizontal gene transfer—probably from a virus—initially hijacked and ultimately usurped the roles and targets of Rb and E2F, thus explaining the conserved G1/S network topology despite divergent transcriptional machinery ^7^.

In both yeast and metazoans, the G1/S regulon comprises genes whose expression is needed for DNA replication in S phase and for further cell-cycle progression ^6, 8– 10^. Moreover, the temporal order of gene activation suggests a prioritization of functions implicated in triggering the positive feedback loop, such as E2F1 and cyclin E in metazoans and Cln1 and Cln2 in budding yeast ^11^. Presumably as a consequence of this and other feedback loops, both systems show ultrasensitivity—the ability to convert graded inputs into all-or-none responses ^12, 13^—and hysteresis—the ability to maintain pathway activation after withdrawal of a mitogenic stimulus ^14^. The bistability underlying G1/S transcriptional activation in all eukaryotes is a feature expected of an irreversible cell fate switch ^15^. Results in both budding yeast and mammalian cells seemed to indicate that cell-cycle commitment and induction of the G1/S transcription program (measured as Whi5 nuclear exclusion or E2F1 promoter activation, respectively) might be one and the same ^12, 14^.

## Making the cell-cycle commitment: the R point, re-defined

There are challenges to this unified view of cell-cycle commitment. Specifically, the nature and timing of the R point—indeed, the very existence of a single discrete point at which cell-cycle commitment occurs—remain open to debate. In part, this reflects the relative difficulty of generalizing from results obtained in different mammalian cell lines, but a more significant source of uncertainty might be the different ways R-point passage has been measured. A sharp transition from mitogen dependence to independence was first detected in quiescent cells stimulated to re-enter the cycle by addition of serum ^1^. This approach necessitated the perturbation of the cell cycle in order to study it, i.e. by initial imposition of a block to proliferation (e.g. serum starvation). This potential problem was addressed in subsequent studies by subjecting asynchronous, continuously cycling populations to serum deprivation or other treatments and tracking individual cells by time-lapse microscopic imaging (seldom discussed is how commonly such successive, uninterrupted divisions occur in multicellular organisms outside of embryonic development or certain highly proliferative tissues). By these methods, the “age” of each cell (i.e. time since the previous mitosis) could be determined and correlated with its response (i.e. whether it arrested in G0 or divided). In Swiss 3T3 mouse fibroblasts, this technique revealed a fairly synchronous transition during G1 from susceptibility to cell-cycle exit upon serum withdrawal to resistance ^2^. This division of G1 into pre- and post-commitment intervals fit with previous definitions of the R point ^1^ and suggested a common mechanism in G0 cells going through a “first” cycle and cycling cells passing directly from mitosis through G1 and back into S phase.

Besides serum starvation, R-point passage in these early studies was shown to be sensitive to amino acid deprivation, inhibition of protein synthesis, or treatments that elevated cellular cAMP levels ^1, 2^, suggesting a common point in G1 at which nutritional, mitogenic, and internal signals were integrated to arrive at the decision to divide. Surprisingly, however, a subsequent time-lapse analysis of asynchronous NIH 3T3 cell populations revealed sensitivity to blockade of mitogenic signaling (i.e. ability to arrest or exit the cell cycle in G1) only in cells microinjected with anti-Ras antibodies *during G2 or mitosis of the previous cycle*, whereas anti-cyclin D1 antibodies retained their ability to block progression until just before S-phase entry ^16^. In contrast, after release from quiescence, cells became refractory to anti-Ras or anti-cyclin D1 antibodies (or to cycloheximide or serum removal) at roughly the same time. Ras activation depends on serum stimulation and is required for cyclin D1 expression ^17^, suggesting that the mitogen-sensing event decisive for R-point passage occurs during G0-G1 progression in cells exiting quiescence but during the previous cycle in continuously dividing cells.

## A cell fate switch determined by Cdk2 levels in G1

Can this concept—that the decision to commence S phase and commit to a round of division is based on mitogen sensing and signaling during the previous G2—be reconciled with earlier studies that seemed to point to G1 as the critical interval? A simple explanation is that quiescent and cycling cells do things differently and that detection of a commitment step in G2 required the analysis of cycling rather than G0-arrested cells. Pieces of cell-cycle machinery that can be re-used in consecutive cycles might be disassembled and/or degraded during quiescence and need to be accumulated *de novo* upon re-entry. This cannot be the whole story, however, because earlier time-lapse analyses of responses to serum deprivation *in cycling cells* placed the R point in G1, approximately 3 hours after completion of mitosis ^2^.

A second explanation recently emerged, together with a possible mechanistic insight into the dichotomous behavior of cycling and quiescent cells. By introducing a fluorescent live-cell sensor of CDK activity and performing time-lapse analysis, Meyer and colleagues uncovered a bifurcation after mitotic exit into discrete cell populations with low or intermediate Cdk2 activity ^18^. MCF10A breast epithelial cells with low Cdk2 entered a transient G0-like state in which they were sensitive to arrest by an inhibitor of MEK (a mitogen-stimulated kinase), whereas those with residual Cdk2 were refractory to MEK inhibition, retained detectable Rb phosphorylation (albeit on a site that was refractory to allele-specific inhibition of Cdk2 in human colon cancer-derived cells ^19^—see below), and could commit to another round of division immediately. Relative distribution between the low- and high-Cdk2 populations was variable among different cell lines and inter-convertible within a population by mitogen deprivation imposed in an 8-hour window ending at mitosis of the prior cell cycle; the low-Cdk2 population that emerged increased in roughly linear fashion as serum was withdrawn at progressively earlier times during this interval. Even the behavior of the Swiss 3T3 cells analyzed by Zetterberg and colleagues ^2^ seemed to be accounted for: this line had a higher fraction (~80%) of low-Cdk2 cells during asynchronous, exponential growth compared to MCF10A (~25%) ^18^, perhaps explaining why the earlier studies placed the R point unambiguously in G1 (or perhaps not: the numbers of Swiss 3T3 cells analyzed in the earlier work should have been sufficient to detect the predicted ~20% of cells that would fail to arrest, according to the new model ^2^).

## To the R point and beyond: parsing CDK roles in G1/S progression

The critical determinant of low- and intermediate-Cdk2 states appears to be the level of the CDK inhibitor (CKI) protein p21; in MCF10A cells lacking p21, the low-Cdk2 population was nearly abolished but could be restored by ectopic p21 expression. Of the known cyclin partners of Cdk2, cyclin E1 or E2 was needed to generate a fraction of cells exiting mitosis with intermediate Cdk2 levels, whereas cyclin A2 knockdown allowed normal bifurcation into low- and intermediate-Cdk2 populations but prevented the further increase in Cdk2 activity that accompanies S-phase entry ^18^. Together with a more recent analysis using the same technology ( ^20^ see below), these results strongly implicate Cdk2 in both the initial commitment to divide and a post-commitment step simultaneous—and possibly synonymous—with S-phase entry. They are thus consistent with results of a chemical-genetic analysis of Cdk2 function in non-transformed human cells. In that study, both copies of wild-type *CDK2* were replaced by *CDK2 ^as^*, which encodes an analog-sensitive (AS) mutant variant sensitized to inhibition by bulky adenine analogs. By reciprocal shifts between permissive and non-permissive conditions for Cdk2 activity (-/+ inhibitor), with or without serum, distinct requirements for Cdk2 were detected at or upstream of the R point (i.e. in the mitogen-sensitive interval of G0/G1) and downstream of the R point (i.e. after mitogens were no longer required) but prior to S-phase entry in *CDK2 ^as/as^* cells released from quiescence ^19^.

A requirement for Cdk2 activity at the R point is consistent with earlier studies in quiescent cells that found a correlation between timing of R-point passage and activating phosphorylation of Cdk2, which was in turn negatively correlated with the levels of the CKI p27 ^21^. Moreover, inhibition of Cdk2 ^as^ in a cancer-derived human cell line ^19^ or in mouse embryonic fibroblasts (MEFs) ^22^ diminished the phosphorylation of Rb specifically on residues previously identified as sites preferred by Cdk2 ^23^. In contrast, inhibition of AS Cdk7—responsible for the activating phosphorylation of Cdk2, Cdk4, and Cdk6—blocked Rb phosphorylation on both Cdk2- and Cdk4/6-preferred sites ^24, 25^. Together, these results support a model in which cyclin D-dependent CDKs can initiate but not complete the phosphorylation of Rb to trigger full induction of the E2F transcriptional program ^26, 27^. Finally, in both non-transformed and cancer-derived *CDK2 ^as^* cells, the requirement for Cdk2 activity was more stringent (i.e. susceptibility to arrest by inhibitory analogs was increased) at limiting mitogen concentrations ^19^. This behavior was consistent with the earlier observations that E2F activation in cells exiting G0 was bistable in response to varying serum concentration and more sensitive to pan-CDK inhibitors when growth factors were limiting ^14, 28^. Therefore, chemical-genetic analyses seemed to support the idea that sequential, site-specific phosphorylation of Rb by cyclin D- and cyclin E-dependent kinases underlies the R point and is both necessary and sufficient to promote the switch from mitogen-dependent to -independent modes of cell-cycle progression ( Figure 1).

## R points of contention

Despite the apparent agreement between studies based on different strategies of CDK inhibition, the precise nature of G1/S regulation in mammalian cells is far from settled. Key aspects of the model were challenged in a provocative study by Dowdy and co-workers ^29^, who found by isoelectric focusing that Cdk4 and Cdk6 activity during G1 resulted in Rb isoforms that were exclusively monophosphorylated, apparently stochastically, on all accessible CDK sites. This monophosphorylation is apparently required for Rb function as a transcriptional repressor, whereas derepression/activation of E2F-dependent transcription occurred only when Rb was hyperphosphorylated in “quantum” fashion by Cdk2 late in G1. These results raise interesting mechanistic questions: for example, how can cyclin D-dependent kinases generate all the monophosphorylated isoforms with no apparent site specificity but never a multiphosphorylated form? It might also be difficult to reconcile this model with 1) chemical-genetic analyses, which seemed to suggest preferences of Cdk2 for specific sites on Rb ^19, 22, 25^; 2) structural studies that illuminated discrete mechanisms—direct and allosteric—by which specific phosphorylations regulate different interactions between Rb and its interacting proteins, including E2F and protein phosphatase 1 (PP1) ^30– 34^; and 3) the sequential activation of E2F target genes during G1/S progression detected in earlier studies ^11, 26^, which were broadly consistent with the structural analyses ^35^.

In another example, analysis by immunofluorescence suggested that detectable cyclin E accumulation (and, by inference, Cdk2 activation and Rb phosphorylation) occurred several hours *after* cells in asynchronous cultures lost their dependence on mitogens ^36^. Moreover, in an experiment similar to the R-point analysis of *CDK2 ^as/as^* cells described above ^19^, the transition to mitogen-independent progression was not blocked by treatment with roscovitine, which inhibits Cdk1, Cdk2, Cdk7, and Cdk9 but not Cdk4 and Cdk6, in human diploid fibroblasts exiting quiescence ^36^. This failure might be explained by the micromolar potency of roscovitine towards its CDK targets ^37^, as opposed to the low-nanomolar IC _50_ values of the bulky adenine analogs used to inhibit Cdk2 ^as^ ^38^. But can the delayed timing of cyclin E accumulation be reconciled with a Cdk2 activity requirement at the R point? One possibility is that the discrepancy reflects another difference between quiescent and cycling cells (which would raise a follow-up question: if not required for R-point passage, why are Cdk2 activity levels in early G1 predictive of the ability to enter the next cycle independent of mitogens ^18^?). Perhaps a more satisfying explanation is offered by an analogy with budding yeast Start, in which the *commitment* to divide is temporally correlated with activation of just a few SBF/MBF target genes, i.e. those involved in positive feedback, but precedes wholesale activation of the G1/S regulon ^11^. Similarly, in single-cell analysis of continuously cycling mammalian cells expressing a fluorescent reporter driven by the *E2F1* promoter, the best *predictor* of whether cells would enter S phase in the presence of drugs that block cell-cycle progression (CDK inhibitors or compounds that prevent the activation of Myc) was the peak amplitude of reporter accumulation, even though that peak occurred long after the commitment was made ^13^. Thus, Cdk2-dependent Rb inactivation (and E2F activation) at a small subset of promoters might be sufficient to drive cells past the R point, but sustained operation of the positive feedback loop might be needed to generate detectable cyclin E immunofluorescence.

## Can a commitment be reversible?

In contrast to the debate over which CDKs do what at the canonical R point, there is general agreement about a Cdk2 requirement at the entry to S phase. In RPE-hTERT cells progressing through G1 after release from contact inhibition, allele-specific Cdk2 inhibition prevented S-phase entry of a fraction of cells that had passed the point of mitogen independence ^19^. A recent study in continuously cycling MCF10A cells treated with an inhibitor of Cdk1 and Cdk2 recapitulated this discrete, post R-point requirement for Cdk2 function at S-phase entry ^20^. Moreover, Meyer and co-workers implicated a specific target of Cdk2 in this transition: the anaphase-promoting complex bound to the substrate recognition subunit Cdh1 (APC ^Cdh1^), an E3 ubiquitin ligase that inhibits S-phase entry and is inactivated by CDK-dependent phosphorylation ^39– 42^ ( Figure 1). Irreversible inactivation of APC ^Cdh1^ required Cdk2-cyclin E, occurred invariably, rapidly, and quantitatively within less than 1 hour of entry to S phase, and displayed bistability; hysteresis was dependent on the APC ^Cdh1^-inhibitor Emi1 ^43^, knockdown of which allowed reactivation of APC ^Cdh1^ by addition of a small-molecule CDK inhibitor ^20^. Interestingly, these results echo earlier studies in budding yeast, which uncovered a requirement for S-phase cyclins in the full inactivation of APC ^Cdh1^ at or around the entry to S phase ^44^.

Therefore, APC ^Cdh1^ inactivation has the characteristics expected of a conserved, irreversible cell-cycle transition, just as the Rb-inactivation/E2F-activation step seems to have ^14^. Remarkably, when cycling cells were exposed to DNA damage, hyperosmotic, or oxidative stress and then deprived of mitogens, there was another bifurcation in their response, depending on whether or not APC ^Cdh1^ had already been inactivated. Prior to that event, they could become quiescent and be stimulated to re-enter the cell cycle by re-addition of mitogens; afterwards, they could not exit the cell cycle but instead underwent checkpoint arrest. Once again, Emi1 was required for hysteresis: in cells depleted of Emi1, APC ^Cdh1^ inactivation could be reversed, not merely prevented, by DNA damage ^20^. The seemingly inescapable conclusion is that, under the right (or perhaps wrong) circumstances, cells can effectively exit the cell cycle and regain their dependence on mitogenic stimulation even after they have activated E2F, but only until they inactivate APC ^Cdh1^, at which point they are fully committed to entering S phase and are dependent on a checkpoint to prevent the replication of damaged DNA.

## Conclusions and prospects: what exactly is the (R) point?

It now seems that the mitogen sensing and signaling needed to commit to a round of division can occur during a temporal window spanning G2, M, and G1 in cycling cells but is collapsed to a single discrete point (or a narrower window) in cells exiting quiescence. When individual cells make that commitment depends on their division history, the conditions under which they are growing, and aspects of cell physiology that can vary stochastically within a population (e.g. p21 levels). Finally, passage of the R point is not the last “decision” a cell can make before completing a round of division; cell-cycle exit remains an option, apparently, until S phase commences.

If passage of the R point is reversible, is it still an R point? Can there be more than one “point of no return” in a single cell cycle? To begin to answer these questions, APC ^Cdh1^ inactivation should be dissected and placed in context of upstream and downstream events. Before discounting or downgrading the role of Rb phosphorylation and E2F activation in the commitment to cell division, however, it is important to remember that the R point was only ever truly defined operationally—as a transition from mitogen dependence to independence in *otherwise unperturbed* cells (either quiescent or cycling). That different mechanisms might operate to regulate cell-cycle commitment and S-phase entry in response to other types of signals (including stress) was explicitly acknowledged in previous models ^6^. There is also experimental evidence that mammalian cells have temporally separate signal transduction pathways for responding to growth factors and nutrients, involving the activation of cyclin D-dependent kinases through Ras/Raf/MEK/ERK-dependent signaling and activation of cyclin E-Cdk2 through mTOR- and PI3 kinase-mediated signaling, respectively (reviewed in 45).

That cells can inactivate Rb, turn on E2F-dependent gene expression, and accumulate active Cdk2—and then undo all those steps and revert to quiescence in the face of stress ^20^—would be a revelation but probably not a paradigm shift (and, it should be noted, these “retrograde” steps can only be inferred at this point). After all, one of the events set in motion by R-point passage—cyclin E expression—is almost certainly required for the inactivation of APC ^Cdh1^. Thus, R-point passage will lead directly to S-phase entry in the absence of intercurrent stresses ( Figure 1). That there are tightly regulated steps taken after the initial decision to divide—and an escape route for the unfortunate (and presumably rare) cell that encounters unanticipated metabolic or environmental threats in the R-point to S-phase interval—is a reminder that the cell-cycle machinery has evolved elaborate, reinforcing mechanisms to safeguard genomic integrity and maintain tissue homeostasis. This realization should prompt us to ask whether other undiscovered roads to quiescence branch off from the main cell division pathway at other bistable “commitment” points.
